# Characterization of proteins secreted by pancreatic cancer cells with anticancer drug treatment *in vitro*

**DOI:** 10.3892/or.2012.2020

**Published:** 2012-09-05

**Authors:** TAKANOBU TAKATA, YASUHITO ISHIGAKI, TAKEO SHIMASAKI, HIDEYUKI TSUCHIDA, YOSHIHARU MOTOO, AKIO HAYASHI, NAOHISA TOMOSUGI

**Affiliations:** 1 Medical Research Institute, Kanazawa Medical University, Uchinada; 2 Department of Medical Oncology, Kanazawa Medical University, Takakura, Hachioji 192-8510; 3 Department of Advanced Medicine, Kanazawa Medical University, Takakura, Hachioji 192-8510; 4 Agilent Technologies Japan, Ltd., Takakura, Hachioji 192-8510; 5 Department of Nephrology, Kanazawa Medical University, Uchinada, Japan

**Keywords:** Panc-1, gemcitabin, secretome, 14-3-3 protein sigma, lactoferrin

## Abstract

Pancreatic cancer is one of the most lethal cancers, with an incidence equaling mortality. It is a heterogeneous group of neoplasms in which pancreatic ductal adenocarcinoma is most common. Pancreatic cancer cannot be cured even if detected early. When treatment is initiated, a suitable method of administration of anticancer drugs must be chosen. Anticancer drugs kill tumor cells. However, side effects including initiation are problematic in anticancer drug therapy. Improved methods for the diagnosis of side effects of pancreatic cancer by using sensitive and specific tumor markers are highly desirable. Therefore, efficient strategies for biomarker discovery are urgently needed. Here, we present an approach based on direct experimental access to proteins released by PANC-1 human pancreatic cancer cells *in vitro*. A two-dimensional (2-D) map and catalog of this subproteome, herein termed the secretome, were established comprising more than 1,000 proteins observed by ‘2-D difference in-gel electrophoresis analysis using cyanine dye’. We investigated 22 spots that were 1.20-fold upregulated and 31 spots that were 0.66-fold downregulated by gemcitabine chloride treatment. Proteins in these spots were identified by nano-high-performance liquid chromatography electrospray ionization time of flight mass spectrometry/mass spectrometry. Most secretome constituents were nominally cellular proteins. By mass spectrometry screening, 14-3-3 protein sigma (14-3-3 σ), protein S100-A8, protein S100-A9, galectin-7, lactotransferrin (lactoferrin, LF) precursor, serotransferrin (transferrin) precursor, and vitamin D binding protein precursor were identified. Western blotting confirmed the presence of 14-3-3 σ and LF. We found that upregulation of 14-3-3 σ was associated with apoptosis, and downregulation of LF was found to suppress tumorigenesis.

## Introduction

Pancreatic cancer is one of the most lethal cancers, with an incidence equaling mortality. It is a heterogeneous group of neoplasmis in which pancreatic ductal adenocarcinoma is the most common form (1).

Pancreatic cancers cannot be cured even if detected early. It is one of the most aggressive and lethal cancers worldwide and is highly resistant to chemotherapy. Over the past decade, gemcitabine (2′,2′-difluorodeoxycytidine, GEM) has been the first-line treatment for advanced pancreatic cancer, but it offers only modest benefit due to pre-existing or acquired chemoresistance (2). Furthermore, recent clinical studies indicate that only 20% of patients with advanced pancreatic cancer responsed to GEM (3). Combining GEM with other chemotherapeutic agents improves the therapeutic outcome, but results to date remain meager. Therefore, novel approaches that significantly enhance the effects of GEM or overcome chemoresistance to the drug are needed to effectively combat pancreatic cancers.

Proteins that are released by human tumor cells *in vivo* and reach the circulation are strongly outweighed by all the normal blood constituents. Thus, seeking an alternative source for the discovery of biomarkers for assessing ‘response to GEM’, we have developed a protocol that provides direct experimental access to a promising subproteome of proteins released by human pancreatic cancer cells *in vitro*. Release of proteins from tumor cells *in vivo* and *in vitro* is due to diverse mechanisms and is not confined to classical secretion, but for the sake of simplicity we follow previous publications and refer to similar subproteomes subproteomes. Classical secretion is the most obvious mode of protein release and is expected to be relevant for proteins such as extracellular matrix molecules. Exosomes are membrane-coated vesicles derived from multivesicular bodies in the late endosomal compartment. They were first detected as products of pancreatic cells and are regarded as important devices for intercellular communication in the regulation of responses to GEM. We have therefore established an empirical approach for the isolation, identification and characterization of the subset of proteins released by pancreatic carcinoma cells treated with GEM *in vitro*. With this aim, we chose the PNAC-1 pancreas carcinoma cell line as a model. Proteins were harvested from conditioned media, concentrated and resolved using two-dimensional difference in-gel electrophoresis (2D-DIGE) and labeled with cyanine (Cy) dye.

Differential analysis showed that, 53 spots in the gel revealed marked differences in protein expression. Twenty-two spots were upregulated >1.2-fold in response to GEM treatment and 31 spots were downregulated <0.66-fold (P<0.01). These spots were picked from other gels which could be assigned to distinct spots in the master gel. Approximately 50 proteins were identified from these spots by nano-high-performance liquid chromatography-electrospray ionization time of flight mass spectrometry/mass spectrometry (HPLC-ESI-MS/MS). Most of them were nominally cellular proteins. The identified proteins included the secreted proteins 14-3-3 protein sigma (14-3-3 σ), protein S100-A8, protein S100-A9, galectin-7, lactotransferrin (lactoferrin, LF) precursor, serotransferrin (transferrin, TF) precursor, and vitamin D binding protein precursor. Western blot analysis confirmed the upregulation of 14-3-3 σ, which is associated with apoptosis, and the dowregulation of LF was found to suppress tumorigenesis.

## Materials and methods

### Chemicals and reagents

Cy dye DIGE fluors (Cy2, Cy3 and Cy5 for minimal labeling), IPG buffer (pH 3.0–10.0), Immobile DryStrip (24 cm, pH 3.0–10.0), sodium dodecyl sulfate (SDS), *N,N,N′,N′*-tetramethylethylenediamine, bind-silane, urea and thiourea were obtained from GE Healthcare (Tokyo, Japan). *N,N*-dimethyformamide (DMF) was purchased from Sigma-Aldrich (Tokyo, Japan). 2-amino-2-hydroxymethyl-1,3-propanediol (Tris-(hydroxymethyl)-aminomethane), potassium hexacyanoferrate (III), sodium thiosulfate, acetonitrile, acetone, dithiothreitol, iodoacetamide and tetrafuloroacetic acid were obtained from Wako Pure Chemical Industries, Ltd. (Osaka, Japan).

SYPRO Ruby was purchased from Invitrogen (Tokyo, Japan). GEM chloride was obtained from Eli Lilly Japan K.K. (Kobe, Japan). The Bradford protein assay kit was purchased from Bio-Rad Laboratories (Tokyo, Japan). Centriplus YM-3 was obtained from Millipore (Bedford, MA, USA).

### Cell culture

The human pancreatic carcinoma cell line PANC-1 was obtained from RIKEN BioResource Center Cell Bank (Japan). PANC-1 cells were maintained in Dulbecco’s modified Eagle’s medium (D-MEM) supplemented with 10% fetal calf serum (FCS), 2 mM glutamine, 100 U/ml penicillin and 100 mg/ml streptomycin. For secretome preparation, cells were cultured at 1.5×10^6^ cell/ml in D-MEM until of 70–80% confluence (4).

### Treatment with GEM

GEM at 10 μg/ml was added to the cells. The cells were incubated for 24 h, then washed five times with phosphate-buffer saline (PBS) and incubated in serum-free medium (Sigma, St. Louis, MO, USA) for another 48 h. This protocol did not measurably influence the apoptosis rate compared with standard culture conditions. GEM exhibits cytotoxicity against cultured PANC-1 cells with an IC_50_ value of 16 μg/ml (3).

### Secretome purification

Conditioned medium was collected from the culture dishes and cooled on ice. Floating cells and cellular debris were removed by centrifugation (2000 × g, 10 min) followed by sterile filtration (pore size, 0.22 μm) (5).

Proteins were concentrated by ultrafiltration using Centriplus YM-3 centrifugal filter devices according to the manufacturer’s instructions. The total protein amount was determined using a standard Bradford protein assay.

### 2-DE and protein labeling with Cy dye

For 2-D gel electrophoresis with Cy dye, to 50 μg protein in medium acetone (20-fold) was added and incubated at −20°C for 2 h. Then, acetone was removed by centrifugation (7000 × g, 5 min) and the precipitation was collected and dried in a SpeedVac (VC-15SP, Titec Co., Ltd., Saitama, Japan). The pellet was resuspended in 40 μl isoelectric focusing (IEF) sample buffer [30 mM Tris-HCl, 8 M urea, 4% (w/v) 3-(3-chloamideopropyl)dimethylammonio)-1-propanesulfonic acid (CHAPS; pH 8.5)]. Cy dye stock (1 nMl/μl) was diluted in anhydrous DMF (Sigma) to final concentration of 400 pM/μl and dye was added per 50 μg protein. Two gels were used, control samples were labeled with Cy3 and samples from GEM treatment were labeled with Cy5 for 2 gels (6). Cy3 and Cy5 were used for the replacement samples for one gel. Protein (25 μg) from control samples and GEM treatment samples were mixed and Cy2 was added to prepare the internal standard. The samples were vortexed, centrifuged for 10 sec, and incubated on ice for 30 min in the dark. The labeling reaction was terminated by adding 1.0 μl L-lysine stock solution (10 mM). Labeled proteins were mixed. Then, 330 μl inhibition buffer [8 M urea, 2% (w/v) CHAPS, 40 mM dithiothreitol (DTT), pH 3.0–10.0, pharmalyte, 1% (w/v) bromophenol blue] was added. We picked up the spot from another 2-D-gel to analyze nano-HPLC-ESI-MS/MS.

Precipitation was performed using 2-D DIGE technology (GE Healthcare). DIGE gels were scanned with Typhoon 9400 Variable Mode Imager (GE Healthcare). Excitation and emission wavelengths were chosen specifically, supernatants was separated by 2-D polyacrylamide gel electrophoresis (PAGE) using immobilized pH gradient (IPG) strips. IPG gel with a linear gradient of pH 3.0–10.0 (24 cm) was used for IEF. The IPG gel was rehydrated for 10 h at 20°C using an IPGphor (GE Healthcare Bioscience). IEF at 20°C was programmed as follows: 1 h at 500 Vh, 1 h at 800 Vh, 3 h at 13.5 Vh, 3.75 h at 20–30 Vh (linear increase) (7). After IEF, the strips were incubated at room temperature for 30 min in a buffer consisting of 1.5 M Tris-HCl (pH 8.8), 6 M urea, 30% (v/v) glycerol, 2% SDS, 16 mM DTT, and 0.002% bromophenol blue (BPB) (6). Then, they were incubated in equilibration buffer containing 2.5 mg/ml iodoacetamide solution (other components were the same as in the solution containing DTT for 30 min) (6).

2-D SDS-PAGE on 10% running gel (24×20×0.15 cm) was performed as described below. The protocol for SDS-PAGE at 20°C was as follows: 20 min at 2.5 w/w gel, 3 h at 20 w/w gel. For each preparative gel, a total of 150 μg protein labeled with Cy and 200 μg non-labeled protein was loaded.

### 2-DE image analysis

DIGE gel image was scanned at 100 μm resolution on Typhoon 9410 variable mode imager (GE Healthcare) using excitation/emission wavelengths specific for Cy2 (488/520 nm, blue laser), Cy3 (532/580 nm, green laser) and Cy5 (633/670 nm, red laser) (6). Laser power was chosen so that no saturated signal was obtained from any protein spot. Resolution was 100 μm. DIGE gels were analyzed using DeCyder 6.5 software (GE Healthcare) in batch processor mode with an estimated number of spots set to 2200 and the spot exclusion filter set to exclude any spot with a volume <7500. A batch processor was used to link the Differential In-gel Analysis (DIA) and Biological Variation Analysis (BVA) modules together in an automated fashion (7–11). The gel containing the highest number of spot features was designated the master gel, and manual spot matching was then performed to correctly match the remaining three Cy2 gel images and the Sypro Ruby stain master. In DIA, spot boundaries and volumes were co-detected for Cy3, Cy5, and Cy2 channels on each gel, and protein spot abundance was expressed as a standard:sample ratio. In BVA, protein abundance was compared across multiple samples using the internal standard to normalize between gels, and statistical analysis was performed to obtain the average ratio and one-way analysis of variance values between samples. The DIA module was used for pair wise comparisons of control and GEM treatment groups with the mixed standard present in each gel and for the calculation of normalized spot volume/protein abundance.

### Protein staining with SYPRO Ruby and Silver nitrate

For 2-D-gel electrophoresis and MS analysis, acetone (20-fold) was added to 200 μg non labeled proteins in medium. After 2 h at −20°C, acetone was removed by centrifugation (7000 × g, 5 min) and the precipitate was collected and dried in the SpeedVac. Two hundred micrograms of protein was loaded on on the gel. The gel was stained with SYPRO Ruby and spots of interest (downregulated spots) were picked with a spot picker (Ettan DIGE Sopt Picker, GE Healthcare). Another 200 μg non-labelled protein on the gel was stained with Silver nitrate and spots of interest (upregulated spots) were picked manually.

### In-gel tryptic digestion

To identify proteins, silver-stained and SYPRO Ruby-stained spots were excised from the gel. They were washed with a solution of 30 mM potassium hexacyanoferrate (III) and 100 mM sodium thiosulfate for 15 min, then washed three times with water. Proteins in the gel were reduced with 10 mM DTT/100 mM NH_4_HCO_3_ (90 min, 56°C) and alkylated with 55 mM iodoacetamide/100 mM NH_4_HCO_3_ (45 min, in the dark at room temperature) (6). Gel spots were washed with acetonitrile and dried in a SpeedVac. Dried gel particles were rehydrated with digestion buffer containing 20 ng/ml sequencing grade trypsin in 100 mM NH_4_HCO_3_ at 0°C for 30 min. Then they were incubated at 37.7°C overnight (6). After digestion, peptides were first extracted from gel pieces with 50% ACN/0.1% tetra fluoroacetic acid (TFA) (50:50), followed by second extracted from gel pieces with 75% ACN/0.1% TFA (75:25).

The two extracts were pooled and concentrated in a SpeedVac, and 0.5% TFA was added to approximate 20 μl of the concentrated solution. Desalting was performed using ZipiTip μC18 (Millipore, Bedford, MA) following the manufacturer’s instructions.

### Identification by mass spectrometry

Tryptic peptides were analyzed by nano-HPLC-ESI-TOF-MS/MS using a nano Frontier LD (Hitachi High Technologies, Ltd.). Peptide identifications were performed using the Mascot search engine. Within the ProteinScape database, protein search was initiated using Mascot search algorithms. Proteins were identified by searching against a human subset of the Swiss-Prot protein database using the Mascot 2.1.0 search algorithm. The following search parameters were selected: up to one missed cleavage site in case of in complete trypsin hydrolysis was allowed and data were searched using carbamidation and oxidation as variable modifications. The peptide mass tolerance was set at 0.5 Da for monoisotopic masses and 0.6 Da for fragment masses. All searches were run in the mammalian protein subdatabase of Swiss-Prot database to exclude putative contamination of bovine serum proteins originating from the culture medium.

Similarly, tryptic peptides were analyzed by another nano-HPLC-ESI-TOF-MS/MS using Agilent 6500 (Agilent Technologies, Ltd.). Peptide identifications were performed using the Mascot search engine (data not shown).

### Western blot analysis

Protein concentrations were determined by the Bradford assay using bovine serum albumin as a standard (Protein Assay kit, Bio-Rad Laboratories). Total protein extracts (50 μg) were mixed with SDS sample buffer (6.25 mM Tris-HCl, pH 6.8, 2.3% SDS, 10% glycerol, 5% β-mercaptoethanol, 0.005% bromophenol blue) and resolved by SDS-PAGE on 10–20% gradient acrylamide gels (8). Proteins (50 μg) were detected immunologically following semidry electrotransfer (Trans-Blot SD semi-dry electrotransfer system, Bio-Rad Lboratories) onto PVDF membranes (Millipore). The membranes were blocked with 5% non-fat dry milk in Tris-buffered saline with Tween-100 for 30 min at room temperature and incubated for 2 h at room temperature with the following primary antibodies: anti-14-3-3 σ (1:1000, Abcam, rabbit monoclonal antibody), and anti-LF (1:5000, Abcam, rabbit monoclonal antibody). After washing three times in 0.5% non-fat dry milk in Tris-buffered saline with Tween-100, blots were incubated with horseradish peroxidase-conjugeated secondary antibody (diluted 1:5000, Abcam) for 2 h at room temperature. Immunoreactive complexes were visualized using HRP-DAB detection kit (Wako). Bands were measured and calculated using LAS-4000 (Fujifilm).

### SDS-PAGE

Proteins in control and GEM treated smples (50 μg) were analyzed by SDS-PAGE. We performed SDS-PAGE in the presence of 2-mercaptoethanol using slab gels in a Tris/glycine buffer system (pH 8.3), as described by Schagger and von Jagow (12). The gel was stained with Coomassie Brilliant Blue.

## Results

### Control media and GEM treated media were filtered and concentrated

**Figure 1:**
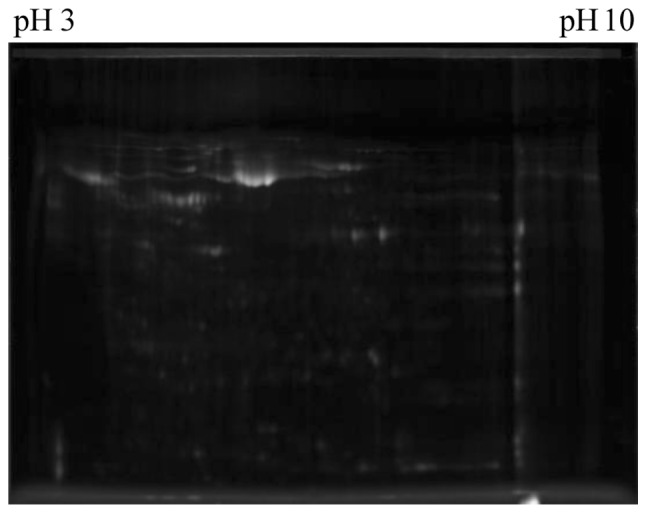
Typical DIGE gel for conditioned medium (CM). Match of all fluorescent Cy spots.

Proteins in control media and GEM treated media were differentially labeled and analyzed by 2-D DIGE. Three replica gels were considered for the quantitative and statistical analysis using the DeCyder™ 6.5 software. This analysis revealed changes in the abundance of 53 spots. Twenty-two spots were significantly upregulated (average. GEM treatment/control ratio >1.2, P≤0.01), whereas 31 were downregulated (average GEM treatment/control ratio <0.66, P≤0.01). Fig. 1 shows a representative 2-D gel image. Arrows indicate proteins identified whose expression was within the 99th confidence level.

**Figure 2:**
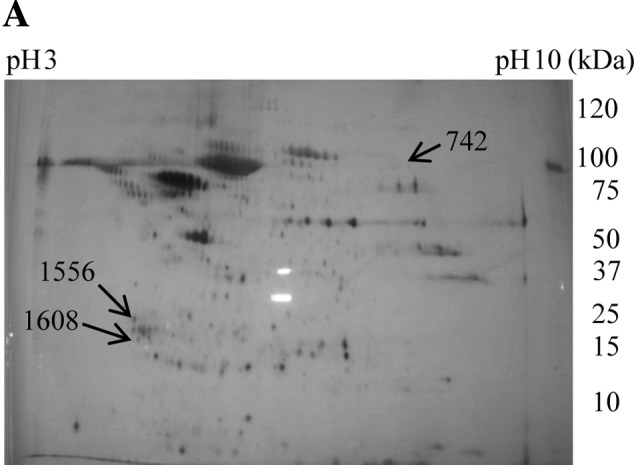

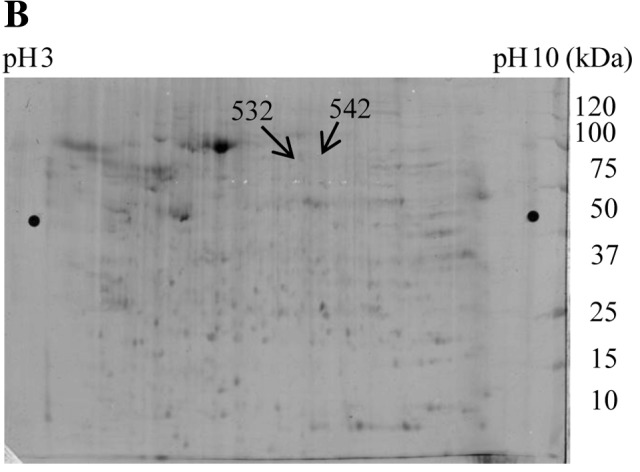
Typical DIGE gel of conditioned medium. (A) The gel was silver stained. Upregulated spots (742, 1552 and 1608) were marked with an arrow on the silver stained gel. (B) The gel was stained with SYPRO Ruby. Downregulated spots (532 and 542) were marked with arrows on the stained gel.

For MS analysis, each 200 μg of non-labeled protein in the medium was subjected to 2-D gel electrophoresis. One gel was stained with silver nitrate and another with SYPRO Ruby. Twenty-two upregulated spots were picked from the gel stained with silver (Fig. 2A, the data of 22 spots are not shown), and 31 downregulated spots were picked from the gel stained with SYPRO Ruby (Fig. 2B, the data of 31 spots are not shown). After in-gel tryptic digestion, protein identifications were combined using the Mascot search engine against the Swiss-Prot database to yield a set of ‘mammalian’ protein identifications with confidence values. Proteins identified as bovine or from another mammalian were removed because of the possibility of contamination from bovine serum albumin. As a result, 37 upregulated and 30 downregulated ‘human proteins’ were identified (data not shown). The subcellular locations of most identified proteins were the cytoplasm, nucleus and membrane. Secreted proteins among the upregulated proteins comprised 14-3-3 σ, protein S100-A8, protein S100-A9 and galectin-7. Secreted proteins among the downregulated proteins comprised LF precursor, TF precursor, and vitamin D binding protein precursor (Table I).

LF precursor consisted of 710 amino acids and produced six proteins or peptides by molecular processing (http://www.uniprot.org/uniprot/P02788) (Table II). The regions of LF precursor identified by nano-HPLC-ESI-TOF-MS/MS were amino acids 191–199, 316–320, 321–328, 424–435 and 542–552 (Table I). We could not identify the true protein using MS/MS data alone. However, the spots on 2-DE gel indicated that the molecular weight was ~60–80 kDa and the pI 8–9 (Fig. 2B).

**Figure 3:**
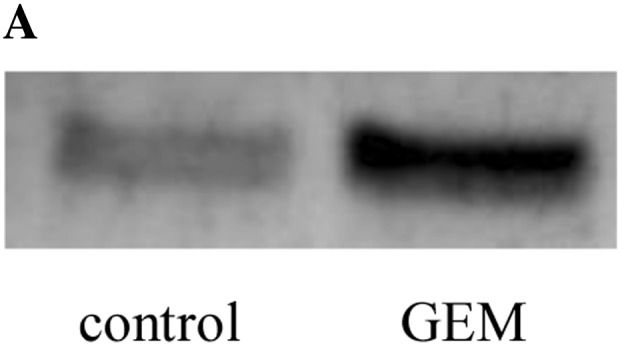

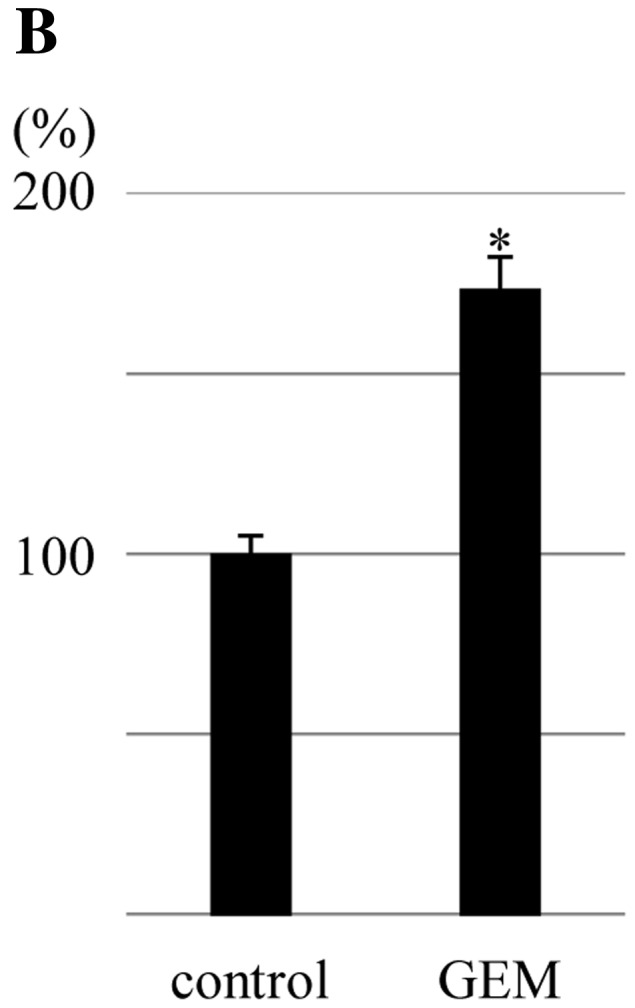
Validation of 14-3-3 σ by western blotting. Fifty micrograms of proteins was applied to the gel. The proteins was transferred to PVDF. Expression of 14-3-3 σ in conditioned medium treated with GEM was upregulated compared with control. (A) Pair of bands from control and GEM treatment. (B) Graph based on the area (n=3). ^*^P<0.05.

**Figure 4:**
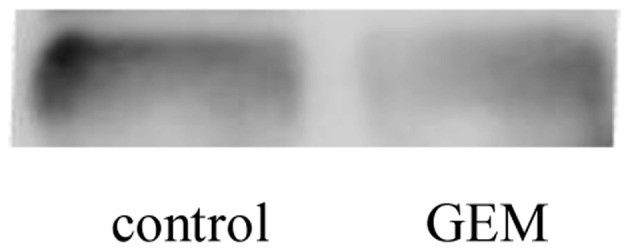
Validation of LF by western blotting. Fifty micrograms of protein was applied to the gel. The expression of LF in control conditioned medium was downregulated compared with GEM treatment medium (n=3).

To validate 14-3-3 σ and LF, we performed western blot analysis to determine the levels of these proteins in control and GEM treated media. In treated medium 14-3-3 σ was upregulated (Fig. 3), but LF was downregulated (Fig. 4). The GEM treatment/control ratios for 14-3-3 σ and LF precursor were 2.87 and 0.38, respectively (Table I). These data were consistent with the data of western blotting.

**Figure 5:**
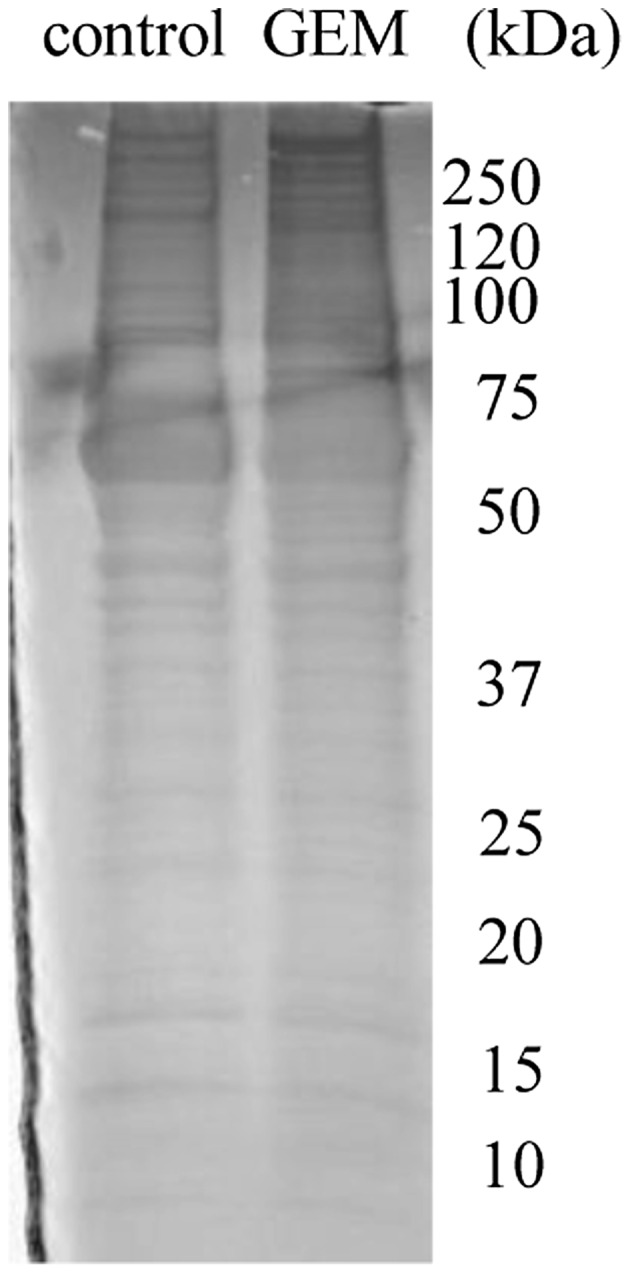
SDS-PAGE analysis of CM. Fifty micrograms of protein was applied to the gel, and the gel was stained with Coomassie Brilliant Blue (n=3). In the regions of 60–8 and 20–30 kDa, control and treatment samples were of equal quantity.

When validation of proteins in lysate was performed, we used β-actin or G3PDH for the reference. However, in the present study proteins in the control and treatment samples were analyzed by SDS-PAGE. The gel was stained with Coomassie Brilliant Blue (Fig. 5).

## Discussion

The mechanism of the anticancer effect of GEM is the inhibition of DNA synthesis. However, information regarding other such events is limited. A transcriptome approach revealed upregulation of 53BP1 mRNA in PANC-1 cells treated with GEM (3). However, use of proteomics in pancreatic cell lines treated with GEM has not been reported. We therefore used secretome analysis to investigate the response of PANC-1 cells treated with GEM. In recent years, studies on secretome have seen rapid acceleration as a result of technological advances, particulary in proteomics.

A method for secretome analysis has been established (5). Carcinoma cells or primary cells were maintained in medium with FBS and incubated for growth. Then, the cells were washed with PBS or serum-free medium (SFM) and incubated in SFM for an appropriate time to remove FBS. Conditioned medium was collected, centrifuged, and subjected to sterile filtration to remove floating cells and cellular debris. Supernatant was collected and concentrated.

Proteins in conditioned medium were thought to be secreted proteins or exported proteins from the cell lines. However, in addition to secreted proteins, the proteins collected from conditioned medium include, cytoskeletal components, membrane components, and nucleus proteins (13,14). This problem cannot be avoided because of cell death. In this study, we targeted secreted protein in the Swiss-Prot database (http://expasy.org/sprot/). However, we were confronted with another difficult problem. In the proteomics analysis of serum, plasma, urine, and organs obtained from humans, protein identifications were combined using a software search engine to yield a set of ‘human’ protein identifications with confidence values. In proteome analysis of human cell lines *in vitro*(15–21), many investigators selected the taxonomic term ‘human’ when using the software search engine to yield a set of protein identifications. However, proteins may be contaminated by bovine proteins originating from FCS (22).

We selected the taxonomic term ‘mammalian’ when using the Mascot search engine to yield a set of protein identifications to exclude bovine proteins. Data from 2-DE and MS screening indicated upregulation of four secreted proteins and downregulation of three secreted proteins in PANC-1 cells treated with GEM. Protein S100-A8, identified from spot 742, might be a pseudo-positive protein because of its high molecular weight and a pI 9–10 (Fig. 2A, Table I).

We confirmed the existence of 14-3-3 σ and LF and the upregulation and downregulation of these protein.

14-3-3 σ belongs to the 14-3-3 protein family (23–25), which is a class of highly conserved proteins involved in regulating signal transduction pathways, apoptosis, adhesion, cellular proliferation, differentiation and survival. Among all 14-3-3 proteins, 14-3-3 σ is the isoform most directly linked to cancer. There are several lines of evidence indicating that 14-3-3 σ acts as a tumor suppressor gene and that its inactivation is crucial in tumorigenesis (26,27). In primary culture of the conjunctival epithelial cell line Cj-ECs, nerve growth factor induced 14-3-3 σ mRNA and protein (28).

Protein 14-3-3 σ is known to be locatd in the cytoplasm and nucleus. Beacuse 14-3-3 σ does not harbor any typical amino-terminal ER export signal, the route of its externalization remains to be determined. However, 14-3-3 σ may be secreted by a non-classial secretory pathway (29). Recombinant 14-3-3 σ was found to sufficiently induce matrix metallolloproteinase 1 (MMP1) expression in fibroblasts (30). It seems possible that GEM induces secretion of 14-3-3 σ. Secreted 14-3-3 σ may act on the cell surface or stimulate cells to suppress tumorigenesis. Altenatively, secreted 14-3-3 σ may be associated with undesirable side effects of GEM.

LF is a member of the transferrin family of iron-binding proteins. It was originally isolated from human milk (31). LF has been detected in many biological fluids as well as in human fetal and adult tissue by radioimmunologic and immunoenzymatic procedures, LF has been detected in many biological fluids as well as in human fetal and adult tissue (32–37). Immunohistochemistry has been used to study the distribution of LF in normal human tissues, such as stomach, kidney, lung, pancreas, liver and bone marrow (34). LF immunoreactivity has been extensively investigated in human neoplastic conditions (38–50). LF inhibited carcinogenesis and metastasis of malignant tumors in mice (51) and in the human pancreatic cell line SPA (52).

If GEM inhibits the secretion or production of LF to promote metastasis, this would be an undesirable side effect.

## Figures and Tables

**Table I:** Identification of secreted proteins picked from spots whose expression was changed by nano-HPLC-ESI-TOF-MS/MS.

Code^a^	Protein	Entry name^b^	Score^c^	M.W.^d^	pI^e^	Peptide sequence^f^	Amino acid number^g^	Cover (%)^h^	Ratio^i^	P-value^j^
532	Lactotransferrin precursor	TRFL	75	78132	8.50	LCAGTGENK	191–199	4	0.41	0.033
						DLLFK	316–320			
						DSAIGFSR	321–328			
						CGLVPVAENYK	424–435			
						YYGYTGAFR	544–552			
	Serotransferrin precursor	TREF	45	77000	6.81	CLKDGADVAFUK	213–225	4	0.44	0.005
						DLLFK	311–315			
						GDVAVK	547–553			
						DLLFR	647–651			
542	Lactotransferrin precursor	TRFL	44	78132	8.50	LCAGTGENK	191–199	4	0.44	0.005
						DLLFK	316–320			
						CGLVPVAENYK	424–435			
						YYGYTGAFR	544–552			
742	Protein S100-A8	S10A8	259	10828	6.51	LLETECPOYIR	37–47	11	0.45	0.033
820	Vitamin D binding protein precursor	VTDB	46	52929	5.40	LCDNLSTK	285–292	1	0.45	0.028
1552	14-3-3 protein σ	1433S	285	27577	4.68	ASLIQK	4–9	31	2.87	0.005
						LAEQAER	12–18			
						YEDMAAFMK	19–27			
						SNEEGSEEKGPEVR	69–82			
						EKVETELQGVCDTVLGLLDSHLIK^k^	86–109			
						EKVETELQGVCDTVLGLLDSHLIK^l^	86–109			
						VETELQGVCDTVLGLLDSHLIK	88–129			
						MKGDYYR	123–129			
						DSTLIMQLLR	215–224			
	Protein S100-A9	S10A9	259	13234	5.71	MSQLER	5–10	64	2.87	0.005
						NIETINTFHQYSVK	11–25			
						LGHPDTLNQEFK	26–38			
						LGHPDTLNQEFKELVR	26–42			
						DLQNFLK	44–50			
						VIEHIMEDLDTNADK	58–72			
						QLSFEEFIMLMAR	73–85			
	Galectin-7	LEG7	41	15066	7.03	SSLPEGIRPGTVLR	8–21	29	2.87	0.005
						LDTSEVVFNSK	55–65			
						GPGVPFQR	76–73			
						HRLPLAR	112–118			
1608	Protein S100-A9	S10A9	45	13234	5.71	NIETINTFHQYSVK	11–25	34	1.20	0.035
						LGHPDTLNQEFKELVR	26–42			
						DLQNFLK	44–50			

a Spot numbers correspond to those included in the 2-D image (Fig. 2. Spot 820 is not shown).

b Swiss-Prot entry name;

c confidence level for identification;

d predicted molecular weight by amino acid sequence;

e predicted pI by amino acid sequence;

f identified amino acid sequence;

g region of identified sequence;

h coverage of identified region in total sequence;

i average GEM treatment/control ratio;

j P-value as calculated by DeCyder 6.5 software;

k this peptide was measured for 2^+^ charged ions;

l this peptide was measured for 3^+^ charged ions.

**Table II:** Moleculare processing of lactotransferrin (LF) precursor.

Name	Sequence	No. of amino acid
No name	1–19	19
Lactotransferrin	20–710	691
Kaliocin-1	171–101	31
Lactoferroxin	338–34	6
Lactoferroxin	543–547	5
Lactoferroxin	680–686	7

LF precursor produced six peptides or proteins.
